# Genomic regions associated with resistance to soybean rust (*Phakopsora pachyrhizi*) under field conditions in soybean germplasm accessions from Japan, Indonesia and Vietnam

**DOI:** 10.1007/s00122-022-04168-y

**Published:** 2022-07-28

**Authors:** David R. Walker, Samuel C. McDonald, Donna K. Harris, H. Roger Boerma, James W. Buck, Edward J. Sikora, David B. Weaver, David L. Wright, James J. Marois, Zenglu Li

**Affiliations:** 1 USDA-ARS Soybean/Maize Germplasm, Pathology and Genetics Research Unit, and Department of Crop Sciences, University of Illinois, Urbana, IL 61801 USA; 2 Department of Crop and Soil Sciences and Institute of Plant Breeding, Genetics and Genomics, University of Georgia, Athens, GA 30602 USA; 3 Department of Plant Pathology, University of Georgia, Griffin, GA 30223 USA; 4 Department of Entomology and Plant Pathology, Auburn University, Auburn, AL 36849 USA; 5 Department of Crop, Soil and Environmental Sciences, Auburn University, Auburn, AL 36849 USA; 6 North Florida Research and Education Center, University of Florida, Quincy, FL 32351 USA; 7 Present Address: Department of Plant Sciences, Sheridan Research and Extension Center, University of Wyoming, Sheridan, WY 82801 USA

## Abstract

**Key message:**

Eight soybean genomic regions, including six never before reported, were found to be associated with resistance to soybean rust (*Phakopsora pachyrhizi*) in the southeastern USA.

**Abstract:**

Soybean rust caused by *Phakopsora pachyrhizi* is one of the most important foliar diseases of soybean [*Glycine max* (L.) Merr.]. Although seven *Rpp* resistance gene loci have been reported, extensive pathotype variation in and among fungal populations increases the importance of identifying additional genes and loci associated with rust resistance. One hundred and ninety-one soybean plant introductions from Japan, Indonesia and Vietnam, and 65 plant introductions from other countries were screened for resistance to *P. pachyrhizi* under field conditions in the southeastern USA between 2008 and 2015. The results indicated that 84, 69, and 49% of the accessions from southern Japan, Vietnam or central Indonesia, respectively, had negative BLUP values, indicating less disease than the panel mean. A genome-wide association analysis using SoySNP50K Infinium BeadChip data identified eight genomic regions on seven chromosomes associated with SBR resistance, including previously unreported regions of Chromosomes 1, 4, 6, 9, 13, and 15, in addition to the locations of the *Rpp3* and *Rpp6* loci. The six unreported genomic regions might contain novel *Rpp* loci. The identification of additional sources of rust resistance and associated genomic regions will further efforts to develop soybean cultivars with broad and durable resistance to soybean rust in the southern USA.

**Supplementary Information:**

The online version contains supplementary material available at 10.1007/s00122-022-04168-y.

## Introduction

Soybean rust (SBR) caused by *Phakopsora pachyrhizi* Syd. is one of the most important foliar diseases of soybean [*Glycine max* (L.) Merr.]. This obligate biotrophic fungus has spread from East Asia to every continent where soybean is grown. After becoming established in South America, it was first observed in the continental USA in November 2004 (Schneider et al. 2005). Severe epidemics can result in yield losses of 70% or more by slowing pod filling and reducing seed weight (Ogle et al. 1979; Kumudini et al. 2008). Although SBR is less of a threat in the USA than it is in parts of South America, it caused > 60% yield loss in unprotected soybean fields in southern Alabama in 2012 (Sikora et al. 2014). In 2013, yield losses of up to 40% occurred in some fields in Alabama and Mississippi (Allen et al. 2014), and statewide yield losses to SBR in Alabama were 4% in 2020 (Allen et al. 2021; Sikora and Conner 2021). While losses can be reduced with timely fungicide applications, this adds to production costs. In addition, extensive use of fungicides in Brazil has diminished the efficacy of some classes of fungicides (Klosowski et al. 2016; Dalla Lana et al. 2018). Soybean cultivars with SBR resistance could therefore play a valuable role in more sustainable disease management strategies.

Resistance to SBR has been reported in *G. max* and other *Glycine* species, including the perennial species *G. tomentella* (Bromfield and Hartwig 1980; Hartman et al. 1992). The appearance of resistance reactions can vary considerably, but reactions to SBR have often been classified into three main infection types (Bromfield et al. 1980). Susceptible plants develop a “TAN” infection type, with two to four uredinia per lesion and profuse sporulation, particularly on the abaxial side of the leaf. A Type 0 infection type is characterized by the absence of macroscopically visible lesions or urediniospores and essentially indicates an immune reaction. Type 0 reactions are uncommon; most resistant soybean hosts have incomplete resistance and develop reddish-brown (RB) lesions, with zero to two uredinia and limited urediniospore production. The intensity of sporulation from both RB- and TAN-type lesions can be influenced by temperature and moisture, as well as by the genotype of the host and aggressiveness of the pathogen (Bromfield 1984).

Race-specific soybean resistance to SBR is conditioned by *Rpp* genes from at least seven reported loci (Bromfield and Hartwig 1980; Childs et al. 2018a; Li et al. 2012). Of these, the *Rpp1, Rpp3,* and *Rpp5* loci are known to have more than one resistance allele (Garcia et al. 2008; Chakraborty et al. 2009; Harris et al. 2015). Most resistance alleles are dominant but recessive alleles have also been reported (Calvo et al. 2008; Garcia et al. 2008). In some cases, the degree of dominance depends on the allele in the susceptible parent (Garcia et al. 2011; Lemos et al. 2011). Due to the extent of pathogenic diversity among *P. pachyrhizi* populations, no *Rpp* gene conditions universal resistance (Bromfield et al. 1980; Bonde et al. 2006). Investigations of the virulence and aggressiveness of *P. pachyrhizi* isolates from the southern USA have revealed extensive pathotype diversity (Pham et al. 2009; Twizeyimana and Hartman 2012; Walker et al. 2011, 2014b). Confirmation that resistance is effective against fungal populations in more than one location and growing season is therefore needed to ensure broad resistance.

Although more than 100 plant introductions (PIs) have been reported to be resistant to *P. pachyrhizi* in the USA, the resistance genes from many of those PIs reside at either the *Rpp1*, *Rpp3* or *Rpp5* locus (Garcia et al. 2008; Pierozzi et al. 2008; Kendrick et al. 2011; Ray et al. 2011; Hossain et al. 2015). Harris et al. (2015) found that SBR resistance genes in 52 out of 75 PIs mapped to the *Rpp3* region on chromosome (Chr) 6 and that 37 of the 52 PIs had a SNP haplotype identical to that of PI 462312, the accession in which the *Rpp3* locus was first discovered. The high percentage of PIs with a resistance allele at one of these loci limits the number of potential resistance gene pyramids, or combinations of *Rpp* genes that could provide broader and more durable resistance (Lemos et al. 2011; Yamanaka et al. 2013; Yamanaka and Hossain 2019).

Prior to the first report of SBR in the continental USA in late 2004, Miles et al. (2006) screened 16,595 PIs from the USDA Soybean Germplasm Collection for resistance to a mixture of *P. pachyrhizi* isolates from Thailand, Brazil, Paraguay, and Zimbabwe. Seedling assays were conducted in a biosafety level 3 greenhouse, and based on the criteria used to define resistance, the authors concluded that 805 accessions (< 4.9%) had resistance to the four isolates. When the 805 PIs were tested in the field between 2006 and 2008, however, only 64 (8%) were confirmed to be resistant to seven southern US populations of the rust fungus (Walker et al. 2011). Furthermore, few of the PIs that Miles et al. (2008) subsequently found to be resistant in South America were also resistant to the US populations (Walker et al. 2011, 2014a, 2014b).

Genome-wide association studies (GWAS) to search for significant genotype–phenotype associations using single nucleotide polymorphism (SNP) markers have been successfully used to understand genetic architecture in panels of germplasm lines and to identify regions of the soybean genome associated with phenotypic variation in traits like resistance to soybean cyst nematode (*Heterodera glycines*), *Phytophthora sojae*, and other soybean pathogens (Guo et al. 2006; Ray et al. 2011; Hwang et al. 2014; Vaughn et al. 2014; Chang et al. 2016; Qin et al. 2017). The GWAS results can also be used to identify germplasm with traits of interest and SNP markers for marker-assisted selection in breeding programs.

Objectives of this study were to (1) evaluate soybean germplasm accessions for resistance to field populations of *P. pachyrhizi* under in the southeastern USA and (2) identify genomic regions associated with soybean rust resistance in those accessions, many of which had been reported to be susceptible to four foreign isolates. Germplasm accessions from a variety of countries and regions were screened initially, but screening was later focused primarily on PIs from Vietnam, southern Japan, or central Indonesia after preliminary evaluations showed that most of the accessions with resistance originated from those three geographic regions.

## Materials and methods

### Materials and experimental design

Two hundred and fifty-six germplasm accessions from Japan, Indonesia, Vietnam, China and several other countries were screened for their reactions to SBR in multiple year-location field tests, along with susceptible controls and PIs with *Rpp* genes at known loci (Tables 1 and S1). Most of the accessions tested were chosen specifically because they had had either susceptible or mixed reactions (i.e., both TAN and RB reactions) to four foreign isolates in earlier greenhouse assays (Miles et al. 2006). After the extent of pathotype diversity of *P. pachyrhizi* populations in different countries became apparent (Miles et al. 2008; Twizeyimana et al. 2008), however, we hypothesized that some of the accessions susceptible to the foreign isolates would have resistance to US pathotypes of the fungus. Each accession tested in a certain year was planted at two locations that year (Table 1). PIs with low-to-moderate disease in the first year they were tested were re-evaluated in the following year to confirm resistance, while PIs that were clearly susceptible were not retested in subsequent years unless they had been reported to have an *Rpp* gene that was effective in South America. The 2008 tests included accessions from more diverse geographical origins and maturity groups (MGs 0 to X) than the tests planted in subsequent years. After 2008, most of the accessions screened originated from southern Japan, Vietnam, or central Indonesia, and were in MGs IV through IX. This was because data from the earlier screenings had shown that few PIs from any other regions were resistant in the southern USA. Seeds were obtained from the USDA Soybean Germplasm Collection in Urbana, IL, USA.

**Table 1:** Locations and years of soybean rust resistance evaluations of plant introductions (PIs)

Location	Geographical coordinates	Year	Principle countries of origin of PIs screened
Attapulgus, Georgia	30°45´ N, 84°29´ W	2008	China, Japan, India, Indonesia, India, Nepal
		2009	Japan, Indonesia, India, Nepal, China
		2012	Japan, Indonesia, Vietnam, China
		2013	Japan and Indonesia
Baton Rouge, Louisiana	30°22´ N, 91°10´ W	2008	China, Japan, Korea, Pakistan
Fairhope, Alabama	30°32´ N, 87°52´ W	2008	Japan, India, Indonesia
		2009	Japan, Indonesia, Vietnam, India, China
Quincy, Florida	30°32´ N, 84°35´ W	2008	China, Japan, India, Indonesia, India, Nepal
		2009	Japan, Indonesia, Vietnam, India, China
		2011	Indonesia, Vietnam, Japan
		2012	Japan, Indonesia, Vietnam, China
		2013	Japan, Indonesia, Vietnam
		2015	Vietnam, Myanmar (2), China (1)
		2016	Vietnam, Japan, Indonesia, China

Susceptible public cultivars representing MGs IV through VII were included in each field tests to provide an indication of SBR disease pressure and development in each year-location environment. Ten susceptible checks were planted in each test, and altogether 16 different susceptible checks were tested during the study. Ten germplasm accessions with known *Rpp* genes were generally planted in each test also to permit detection of possible pathotype differences among *P. pachyrhizi* populations in different years and locations. PIs carrying the *Rpp1* through *Rpp6* genes were planted, including *Rpp1*, *Rpp2* and *Rpp5* accessions with different resistance alleles. The Japanese cultivar Hyuuga (PI 506764), which has resistance alleles at the *Rpp3* and *Rpp5* loci (Kendrick et al. 2011), was also included in each field test.

Field tests were conducted in the southeastern USA between 2008 and 2016 (Table 1). The field tests were planted in at least two locations most years, and usable SBR reaction data were obtained from 14 different year-location environments. Screening nurseries were planted at the University of Florida’s North Florida Research and Education Center in Quincy, Florida, and at the University of Georgia Attapulgus Research and Education Center in southwestern Georgia every year of the study. Tests were also planted at Auburn University’s Gulf Coast Research and Extension Center in Fairhope, Alabama, in 2008 and 2009, and at Louisiana State University’s Central Research Station in Baton Rouge in 2008.

The field tests in Georgia were planted in mid-July, and the tests at most other locations were planted in early to mid-August to delay flowering until precipitation levels became more favorable for *P. pachyrhizi* and disease development later in the growing season. The field plots consisted of single rows arranged with a randomized complete block design, usually with two replications. Depending on the test site, rows ranged from 1.2 to 2.0 m in length, with 0.9 m between rows. Planting and field management methods used for the 2008 to 2013 tests were identical to those described in Walker et al. (2011, 2014b), and the 2015 and 2016 tests in Quincy, FL, were conducted the same way as earlier tests at that location. In Attapulgus, GA artificial lighting was used during the nighttime for one month after emergence of seedlings to delay plant maturation, and agricultural streptomycin was applied to the plots there to reduce the incidence of bacterial pustule (*Xanthomonas axonopodis* pv. *glycines*), as described in Walker et al. (2011).

### Rating of reactions to SBR in field evaluations

Infection with *P. pachyrhizi* at the SBR screening nurseries occurred naturally through infection with urediniospores from nearby soybean fields and patches of kudzu (*Pueraria lobata*). SBR disease severity was rated either on plants in the field, or on leaflets collected from test plots between mid-October and early December, when SBR symptoms and signs (i.e., urediniospores) were visible on susceptible checks. At least 10 leaflets were collected randomly from the mid-canopy, placed in labeled bags, and kept in a refrigerator until they could be examined with a compound microscope. Reactions to SBR in some test locations were evaluated using different rating methods and scales in 2008, when tests were grown in four different states. The scales and methods used at each location between 2008 and 2011 were described in Walker et al. (2014b), and the methods used at Quincy in 2011 were also used for the 2013 to 2015 assays. The inclusion of rating data from susceptible checks helped to mitigate differences in rating methods and persons. Data from a test site were used only if SBR severity and sporulation were high on plants known to be susceptible.

For both severity and sporulation, higher rating values indicated more disease. SBR severity was rated on plants at each nursery location, usually on a scale of 1–5. A 1–9 scale was used at Baton Rouge in 2008, however, and the 2008 test in Fairhope, AL was rated once using a 0 to 8 scale, as described in Walker et al. (2011, 2014b). The ratings from the longer scales were subsequently converted to a 1 to 5 scale so that those ratings could be included in the calculation of the best linear unbiased predictor (BLUP) value for each of the soybean PIs with rating data from multiple locations. For SBR severity, a rating of 1 meant that no rust lesions were visible, while a rating of 5 indicated that the density of SBR lesions on plants or leaf samples was as high as that observed on susceptible checks from the same field test. In Quincy, FL and in some other nurseries, sporulation (i.e., accumulation of urediniospores on the abaxial side of infected leaves) was also rated on a 1 to 5 scale in which 1 meant that no urediniospores were visible, and 5 indicated profuse sporulation, equivalent to that seen on leaves of the susceptible checks. When rating data for both sporulation and SBR severity were collected, a rust index (RI) value was calculated from the two types of ratings as described in Walker et al. (2014a, b). The RI rating was the square root of the product of the severity and sporulation ratings, so it was also in the range of 1.0–5.0.

### Genotype data and statistical analyses

SNP marker data from 285 accessions that had previously been genotyped with the Illumina SoySNP50K Beadchip (Song et al. 2015) were downloaded from the SoyBase (Grant et al. 2010). SNPs with a minor allele frequency (MAF) less than 0.05 were filtered from the dataset, leaving 31,114 SNPs that were used to analyze population structure and carry out the GWAS.

To assess the degree of population structure, principal coordinate analysis (PCoA) was conducted in the GAPIT Version 3 R package (Wang and Zhang 2020). Eigenvalues from the GAPIT output were used to calculate the variation explained by each principal coordinate (PC). A scatter plot of the first two PCs was created with the ggplot2 R package (Wickham 2016). A neighbor joining tree was calculated in TASEL 5 (Bradbury et al. 2007) and visualized with FigTree Version 1.1.4 software (http://tree.bio.ed.ac.uk/software/figtree/). The 20 accessions with the lowest disease ratings (all with BLUPs less than − 0.756) were labeled in the PC plot to facilitate the assessment of the importance of geographic sources of resistance in the panel. Accession names in the PCoA plot and neighbor joining tree were colored based on the country of origin of each PI to make visual assessment more informative.

BLUP values were calculated from the disease ratings of PIs using JMP Pro 14 (JMP, SAS Institute Inc., Cary, NC, USA), which permitted us to overcome the limitations of an incomplete dataset and the differences among test environments. BLUP values were calculated by treating genotype, environment, and genotype × environment interactions as random variables using the Standard Least Squares and restricted maximum-likelihood (REML) methods (Shaw 1987). The BLUP values were then used in the GWAS analysis to identify genomic regions associated with SBR resistance, similarly to the methods described by Steketee et al. (2020).

A GWAS analysis was conducted using the Fixed and random model Circulating Probability Unification (FarmCPU) method (Liu et al. 2016a, b), which eliminates some false positives by running iterations of a fixed-effect model to test markers and a random effects model to define kinship using multiple associated markers. The first three principal coordinates were included in the GWAS model to account for covariance between population structure and phenotype. To determine significant SNPs and correct for multiple testing, the false discovery rate (FDR) for each marker was included in the FarmCPU output. SNPs with an FDR less than 0.05 (*p* < 1.445 × 10^–5^) were considered significant.

## Results

### Disease evaluations

Informative data were obtained from field evaluations in 2008, 2009, 2011, 2012, 2013, 2015, and 2016. Altogether, 256 PIs with unknown or inconclusive resistance to US *P. pachyrhizi* populations were screened in at least two different year-location environments. Disease data were also collected on the 16 susceptible check cultivars and 16 accessions with mapped *Rpp* resistance genes. We added PI 605823 to the latter group after the discovery of the *Rpp7* gene locus. In fact, we developed the populations that Childs et al. (2018b) used to map *Rpp7* after early data from this study showed that PI 605823 was resistant in our field test locations.

SBR severity and urediniospore production on susceptible checks demonstrated that disease pressure was generally high by the time the PI reactions were rated in each test. Rust index (RI) ratings for susceptible checks were between 3.5 and 5.0 in most tests, resulting in positive BLUP values that were often higher than 1.00 (Table 2). In contrast, RI ratings on highly resistant *Rpp* gene differentials like PI 200492 (*Rpp1*) and PI 567102B (*Rpp6*) were typically between 1.0 and 2.0, and BLUP values less than − 1.00. Differences in the reactions of some accessions with known *Rpp* genes between test environments indicated pathotype variation among some of the *P. pachyrhizi* populations.

**Table 2:** Soybean rust on the 20 soybean plant introductions (PIs) with the lowest (negative) BLUP values, six differentials with known *Rpp* resistance alleles, and five susceptible checks

Plant introduction	Name	BLUP value^a^	Origin	Comments
PI 567104B	MARIF 2769	− 1.46	East Java, (central) Indonesia	*Rpp6*
PI 200492	Komata	− 1.33	Shikoku, (southern) Japan	*Rpp1*
PI 567090	MARIF 2688	− 1.24	East Java, (central) Indonesia	
PI 567102B	MARIF 2767	− 1.16	East Java, (central) Indonesia	*Rpp6*
PI 200532	Shiro Hanasaki No. 1	− 1.13	Shikoku, (southern) Japan	
PI 635999	‘DT 2000’	− 1.09	Taiwan/Vietnam	*Rpp3* and *Rpp4*
PI 567061	MARIF 2657	− 1.09	(unknown), Indonesia	
PI 605823	-	− 0.97	Ha giang, (northern) Vietnam	*Rpp7*
PI 200547	Waka Shima	− 0.92	Shikoku, (southern) Japan	
PI 566984	MARIF 2532	− 0.89	(unknown), Indonesia	
PI 566975	MARIF 2521	− 0.84	East Java, (central) Indonesia	
PI 416806	Aso Aogari	− 0.83	Kyūshū and Okinawa, (southern) Japan	
PI 567046A	MARIF 2627	− 0.83	Central Java, (central) Indonesia	
PI 423959	Asomusume	− 0.83	Kumamoto, (southern) Japan	
PI 567034	MARIF 2607	− 0.81	Central Java, (central) Indonesia	
PI 200487	Kinoshita	− 0.80	Shikoku, (southern) Japan	*Rpp5* allele
PI 566982	MARIF 2528	− 0.79	(unknown), Indonesia	
PI 416826A	Cha sengoku 81	− 0.78	(unknown), Japan	
PI 417085	Kumaji 1	− 0.78	Kyūshū, (southern) Japan	
PI 567025A	MARIF 2592	− 0.75	(unknown), Indonesia	
PI 423970	Oshoku akidaizu	− 0.68	Kumamoto, (southern) Japan	*Rpp4* allele
PI 462312	‘Ankur’	− 0.66	Uttar Pradesh, India/Florida, USA	*Rpp3*
PI 506764	‘Hyuuga’	− 0.64	Kyūshū, (southern) Japan	*Rpp3* + *Rpp5* allele
PI 471904	Orba	− 0.61	Java, Indonesia	*Rpp5* allele
PI 417125	Kyūshū 31	− 0.54	Kyūshū and Okinawa, (southern) Japan	*Rpp2*
PI 567068A	MARIF 2666	− 0.47	East Java, (central) Indonesia	*Rpp6* allele
PI 230970	-	− 0.32	(unknown), Japan	*Rpp2* (differential)
PI 459025B	(Bing nan)	0.12	Fujian, (southeastern) China	*Rpp4* (differential)
PI 594760B	(Gou jiao huang dou)	0.77	Guangxi, China	*Rpp1* allele (differential)
PI 567099A	MARIF 2740	0.83	East Java, (central) Indonesia	*Rpp3* allele (differential)
PI 200456	Awashima Zairai	0.97	Shikoku, (southern) Japan	*Rpp5* allele (differential)
PI 200526	Shiranui	1.24	Shikoku, (southern) Japan	*Rpp5* allele (differential)
PI 548986	‘Brim’	0.85	North Carolina, (southeastern) USA	Susceptible check
PI 595645	‘Benning’	1.07	Georgia, (southeastern) USA	Susceptible check
PI 612157	‘Prichard’	1.60	Georgia, (southeastern) USA	Susceptible check
PI 615582	‘Caviness’	1.40	Arkansas, (south-central) USA	Susceptible check
PI 641156	‘NC-Raleigh’	1.27	North Carolina, (southeastern) USA	Susceptible check

^a^Lower (i.e., negative) best linear unbiased predictor (BLUP) values indicate less disease and therefore greater resistance to soybean rust

BLUP values calculated from the disease ratings ranged from − 1.46 (least disease) to 1.60 (most disease). One hundred and thirty-eight accessions had BLUP values of − 0.10 or lower, and the seven most resistant accessions had BLUPs less than − 1.00 (Tables 2 and S1). The nine lines with the least disease were PI 567104B (*Rpp6*), PI 200492 (*Rpp1*), PI 567090, PI 567102B (*Rpp6*), PI 200532, PI 567061, PI 635999, PI 605823 (*Rpp7*) and PI 200547 (Table 2). PI 635999 is the Vietnamese cultivar ‘DT 2000’, which has alleles at the *Rpp3* and *Rpp4* loci. Of the 99 accessions with BLUP values lower than -0.40, 44 were from Japan, 21 were from central Indonesia (mainly Java), and 24 were from northern Vietnam. Among the few resistant accessions not from those areas were PI 518295 from Taiwan and PI 476905A from an unknown location in China. Of the other 23 accessions from China, only PI 594796 and PI 594742 had negative BLUP values, and those were only − 0.07 and − 0.01, respectively (Table S1).

Other effective genes included the *Rpp3* gene in PI 462312, the allele(s) of *Rpp2* in PI 230,970 and PI 417125, the *Rpp5* alleles of PI 200487 and PI 471904, and at least one of the two *Rpp* genes in ‘Hyuuga’ (PI 506764) (Table 2). The *Rpp4* gene from PI 459025B was not effective against most of the *P. pachyrhizi* populations. Other highly resistant accessions with no known *Rpp* genes included PI 567090, PI 567061, PI 566984, PI 566975 and PI 567046 from central Indonesia; and PI 200532, PI 200547 and PI 416806 from southern Japan (Table 2).

Accessions known to have resistance at the *Rpp1*, *Rpp3*, *Rpp4* and *Rpp5* loci showed wide variation in the amount of disease that they developed, indicating different resistance alleles (Tables 2 and S1). Unlike the highly effective *Rpp1* gene of PI 200492, the *Rpp1* allele in PI 561356, the *Rpp1-b* gene from PI 594538A, and the *Rpp1* allele(s) of PI 594760B and PI 594767A did not protect plants against the pathotypes encountered. The *Rpp3* allele in PI 567099A was also ineffective compared to the *Rpp3* gene in PI 462312. The *Rpp5* allele(s) in PI 200487 from southern Japan and in PI 471904 from East Java provided resistance, whereas the *Rpp5* allele from PI 200526 and the recessive *rpp5* allele from PI 200456 did not suppress disease in those two Japanese accessions (Table S1). The *Rpp4* allele that Harris et al. (2015) detected in PI 605791A also conditioned resistance, unlike the allele of the *Rpp4* gene of PI 459025B (Silva et al. 2008). *Rpp6* alleles in PI 567068A, PI 567076 and PI 567129 conditioned resistance but were less effective than the original *Rpp6* gene(s) in PI 567102B and PI 567104B. At least five PIs with single *Rpp* genes had less disease than the cultivar Hyuuga (PI 506764), which has resistance alleles at the *Rpp5* and *Rpp3* loci (Table 2). It is possible that the *Rpp5* allele in Hyuuga contributed little to resistance against the fungal pathotypes encountered.

### Population structure in the germplasm panel

**Fig. 1:**
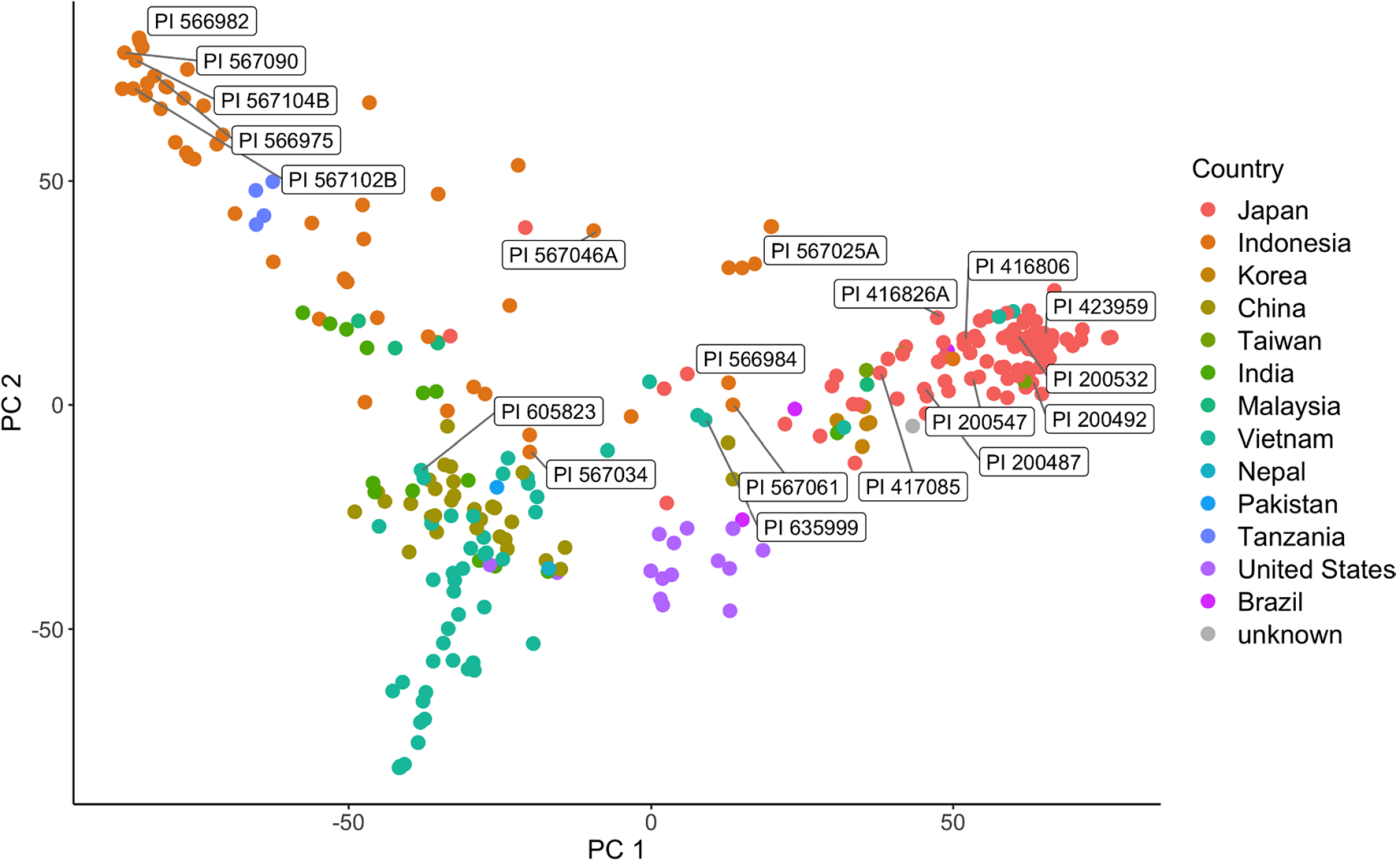
Plot of principal coordinate analysis for a panel of soybean accessions evaluated for their reactions to soybean rust in the southeastern USA. Dots representing plant introductions (PIs) are color-coded based on country of origin. Clusters of PIs from Japan, Indonesia and Vietnam are mostly independent from one another, and the susceptible checks from the USA also formed an independent cluster

**Fig. 2:**
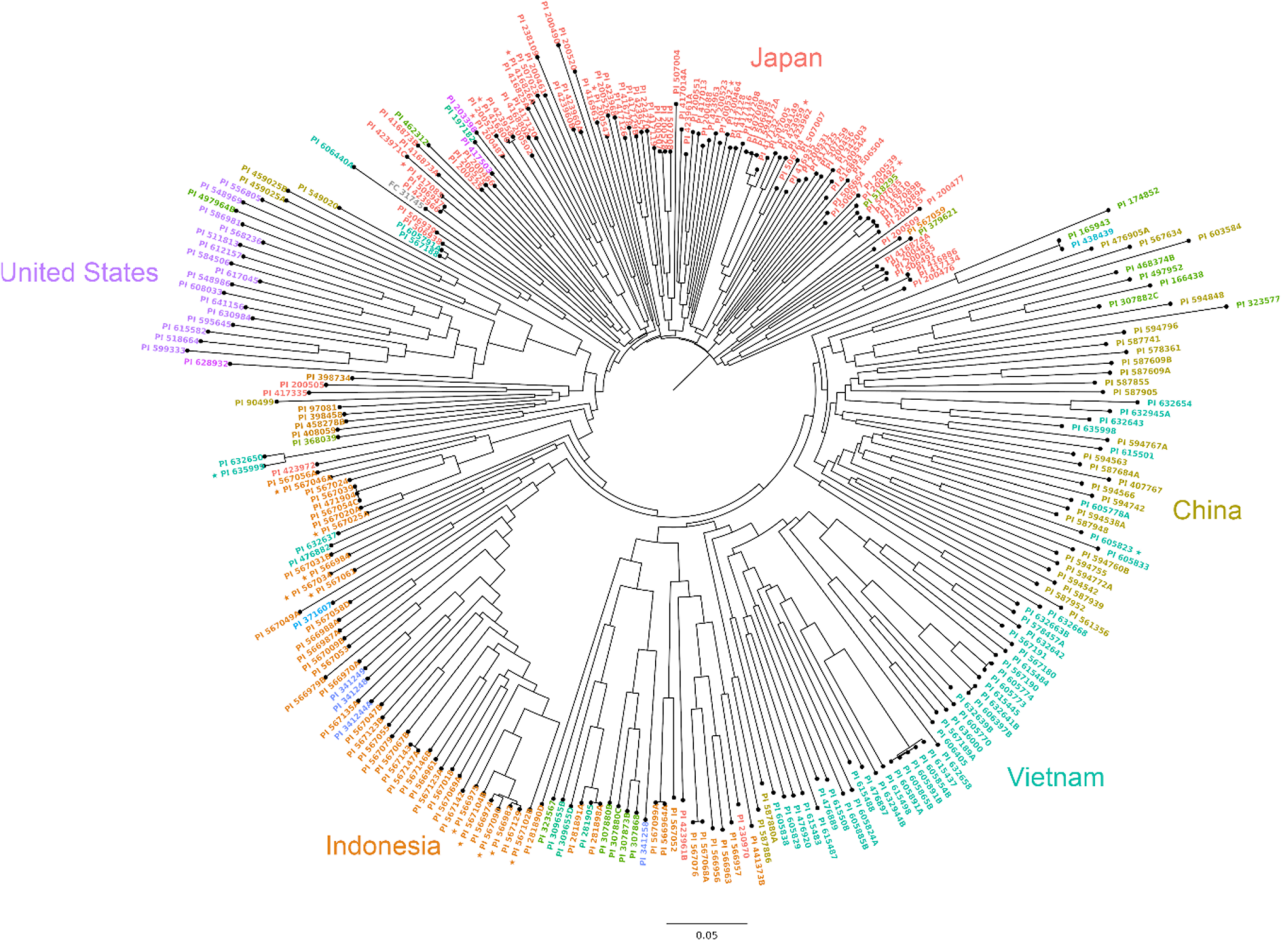
Dendrogram depicting the genetic relationship among soybean plant introductions in a panel of germplasm accessions evaluated for their reactions to soybean rust in the southeastern USA. The grouping patterns also indicate that the majority of the accessions from Vietnam, Indonesia and especially Japan formed distinct groups based on country of origin. The genetic similarities among US cultivars used as checks in the disease assays are also evident

The principal coordinate analysis (PCoA) (Fig. 1) and the dendrogram generated by the neighbor-joining analysis (Fig. 2) revealed population structure in the panel of germplasm accessions. In the color-coded scatter plot of the first and second PCs, clustering of lines was strongly influenced by geographical origin, with separation between most of the PIs from Japan, Indonesia, and Vietnam (Fig. 1). The 20 most-resistant accessions, based on their low BLUP values, showed a strong degree of clustering along PC 1. Ten of these accessions were from Java, eight were from southern Japan, and two were from northern Vietnam. In the neighbor-joining tree, most of the accessions from southern Japan formed a cluster that was separate from the PIs from Indonesia, Vietnam and China (Fig. 2). The majority of the accessions from Indonesia, China and Vietnam also formed distinct clusters, though there were occasional exceptions to this origin-based clustering, especially for some accessions from Vietnam. The presence of population structure was therefore accounted for in the GWAS model.

### Genomic regions associated with resistance to SBR

**Fig. 3:**
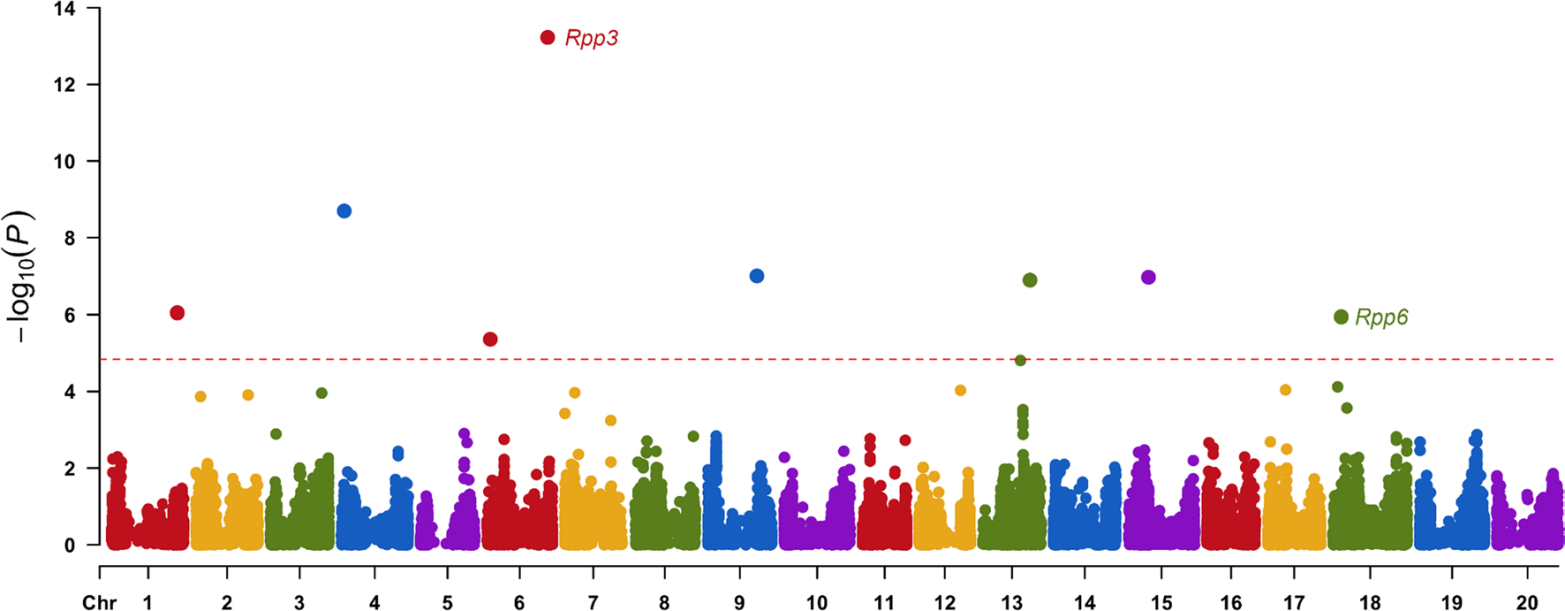
Manhattan plot generated from a genome-wide association analysis of a panel of soybean accessions evaluated for their reactions to soybean rust in the southeastern USA. The X-axis shows the location of SNPs along each chromosome in the genome, and the Y-axis shows the − log_10_ of the *p*-values. The significance threshold was − log_10_(*P*) = 4.84

**Fig. 4:**
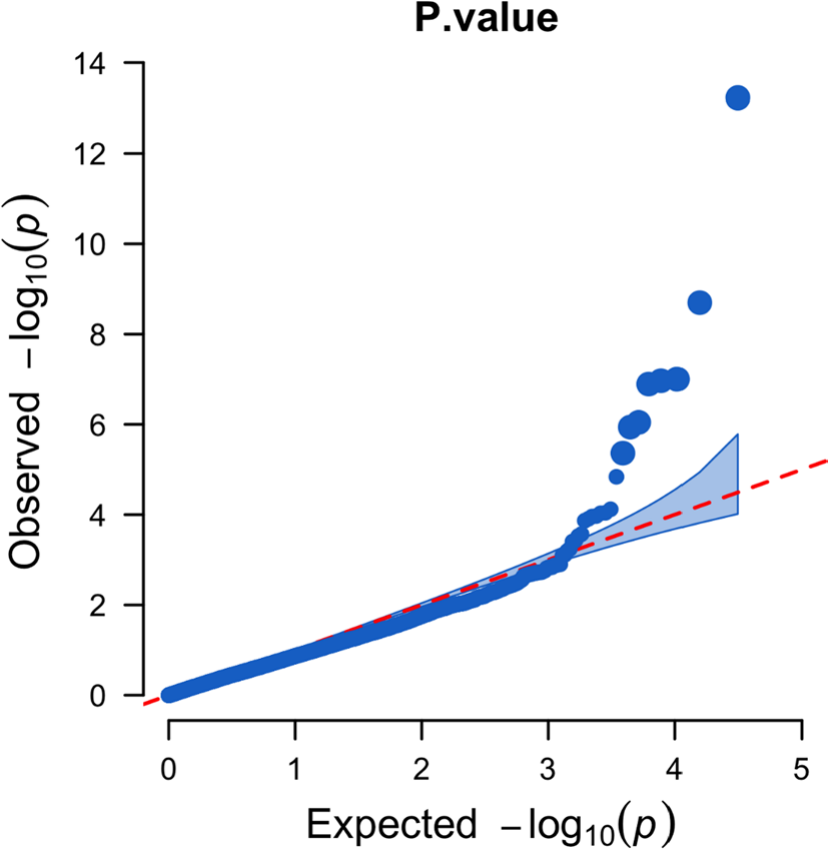
Quantile–quantile (QQ) plot of expected vs. observed *p*-values for each SNP marker used in the GWAS analysis

GWAS analysis using the BLUP disease rating values and 31,114 SNP markers resulted in the detection of eight significant SNPs in eight genomic regions on seven chromosomes (*P* < 1.445 × 10^–5^; − log_10_(*P*) = 4.8) (Fig. 3; Table 3). Single genomic regions were detected on Chrs 1, 4, 9, 15, and 18, and two distinct regions were found on Chr 6 (Fig. 3). The significant SNPs were also evident in the quantile–quantile (QQ) plot of the expected vs. observed *p*-values for the SNPs used in the GWAS (Fig. 4). The effect of the favorable allele at the significant SNPs (i.e., the allele in most of the resistant PIs) ranged from − 0.24 to − 0.10 (Table 3). The significant SNP marker with the highest significance level (ss715594707) was located at position 47,460,008 on Chr 6 in the Wm82.a2 reference assembly, which is approximately 2–3 Mb from the estimated location of the *Rpp3* locus (Fig. 3; Hyten et al. 2009). The *R*^2^ value for this region was 0.23, considerably higher than those of the other genomic regions, which ranged from 0.02 to 0.08 (Table 3). SNP marker ss715594707 had a MAF of 0.25 in the panel. A second significant region on Chr 6 (ss715594035) was detected at position 2,748,236, a region not previously reported to be associated with SBR resistance, perhaps because its R^2^ contribution was only 0.02.

**Table 3:** SNP markers from genomic regions significantly associated with resistance to soybean rust in the field (2008–2016)

SNP ID	Chromosome	Position^a^	Favorable Allele^b^	Unfavorable Allele	− log_10_(*P*)	FDR^c^ adjusted *P*-value	*R*^2^	MAF^d^	Effect^e^
ss715594707	6	47,460,008	T	C	13.23	1.85 × 10^–9^	0.23	0.25	− 0.202
ss715587420	4	2,326,304	T	C	8.70	3.12 × 10^–5^	0.07	0.13	− 0.192
ss715603735	9	38,393,747	A	G	7.00	7.98 × 10^–4^	0.08	0.17	− 0.161
ss715621005	15	15,694,109	A	G	6.97	7.98 × 10^–4^	0.02	0.08	− 0.188
ss715615821	13	36,896,763	G	A	6.89	7.98 × 10^–4^	0.06	0.45	− 0.103
ss715580160	1	51,732,040	A	G	6.05	4.67 × 10^–3^	0.04	0.06	− 0.238
ss715632525	18	6,406,710	G	T	5.94	5.10 × 10^–3^	0.05	0.14	− 0.134
ss715594035	6	2,748,236	A	G	5.36	1.69 × 10^–2^	0.02	0.32	− 0.100

^a^Physical position (in base pairs) according to Wm82.a2 reference assembly

^b^Base at SNP marker associated with lower BLUP values (i.e., higher resistance to SBR). A causative effect on resistance is not implied

^c^False discovery rate

^d^Minor allele frequency

^e^Effect of favorable allele on decreasing BLUP values calculated from disease ratings

Besides the two regions detected on Chr 6, the markers with the highest − log_10_(*P*) values, in descending order of probability, were on Chrs 4, 9, 15, 13, 1, and 18 (Table 3). The effects of the favorable alleles at the SNP markers in those genomic regions ranged from − 0.192 for the marker on Chr 4 to − 0.134 for the marker on Chr 18 (Table 3). The Chr 18 marker, ss715632525, is in a region of Chr 18 that contains the *Rpp6* locus discovered in PI 567102B and PI 567104B (Li et al. 2012; Liu et al. 2016a, b) (Tables 2, 3 and S1). No *Rpp* loci have been reported previously in the other five genomic regions with significant markers.

## Discussion

This study demonstrated that (1) numerous soybean PIs previously reported to be susceptible to *P. pachyrhizi* isolates from other continents were actually resistant to field populations of *P. pachyrhizi* in the southern USA; (2) the majority of the resistant accessions originated from either Japan, Vietnam or Indonesia; and (3) GWAS analysis of BLUP values calculated from disease reactions in multiple years and locations could be used to identify eight genomic regions putatively associated with resistance to SBR. Six of those regions have not previously been reported as far as we know.

*Rpp* genes and genomic regions of the soybean genome associated with resistance to SBR have been identified in previous studies using biparental molecular mapping, bulked segregant analysis or GWAS with seedling reaction data from greenhouse assays (Chang et al. 2016; Garcia et al. 2008; Harris et al. 2015; Hyten et al. 2007, 2009). In most of those assays, plants were inoculated with an isolate collected in a single location and growing season. To our knowledge, this is the first GWAS of SBR resistance that used field data from multiple years and locations, and it is only the second GWAS study for SBR resistance. Unlike greenhouse assays, in which seedling reactions are typically classified into three infection types two weeks after inoculation, our plant reaction data provided assessments of the resistance of adult plants to different field populations of the rust fungus based on sporulation intensity and disease severity. Calculation of BLUP values from the semi-quantitative disease ratings allowed us to obtain estimates of the relative resistance of the germplasm accessions from an unbalanced data set collected in multiple locations over several years. GWAS analysis of the BLUP values made it possible to identify regions of the soybean genome associated with resistance to *P. pachyrhizi* pathotypes prevalent in the southern USA, including regions containing the *Rpp3* and *Rpp6* resistance genes.

The disease ratings demonstrated SBR resistance in at least 132 germplasm accessions that Miles et al. (2006) had previously reported to be susceptible or only partly resistant to four foreign *P. pachyrhizi* isolates. This was less surprising after the extent of pathotype diversity among and within *P. pachyrhizi* populations became evident (Pham et al. 2009; Twizeyimana et al. 2009; Akamatsu et al. 2013). The BLUP values calculated from the disease rating data indicated the relative levels of resistance or susceptibility of the accessions, and several of the PIs with the lowest BLUP values also had high levels of resistance in previous field and greenhouse tests conducted in the southern USA (Walker et al. 2011, 2014a, 2014b). The reactions of some of the PIs also showed that pathotypes of *P. pachyrhizi* populations from fields in the southern USA differ from some South American pathotypes (Garcia et al. 2008; Miles et al. 2008). This illustrates the importance of verifying that soybean germplasm has resistance to local and regional populations of the rust fungus.

*Phakopsora pachyrhizi* populations have high levels of pathogenic diversity within and among populations (Yamaoka et al. 2002; Akamatsu et al. 2013; Twizeyimana and Hartman 2012). It is therefore important to identify germplasm with *Rpp* genes that condition resistance to local pathotypes across years and locations. Data from Stone et al. (2022) reaffirmed that the pathotypes of the Fort Detrick isolates used by Miles et al. (2006) are very different from pathotypes in US *P. pachyrhizi* populations. After finding that only about 50 of the 805 PIs that Miles et al. (2006) selected were resistant to *P. pachyrhizi* pathotypes in the southern USA (Walker et al. 2011), we hypothesized that some accessions with susceptible or mixed reactions to the four foreign isolates would have resistance to at least some US pathotypes. This study proved that hypothesis to be correct; 84 accessions that Miles et al. (2006) reported to be susceptible to SBR had BLUP values ≤ − 0.10 and can thus be considered resistant.

Soybean cultivars with pyramids of two or more *Rpp* genes are likely to have broader and more durable resistance than cultivars with single *Rpp* genes (Yamanaka et al. 2013; Yamanaka and Hossain 2019). Since many of the known resistant soybean accessions have *Rpp* genes at the same loci, however, it is important to continue searching for additional *Rpp* loci and novel alleles. We focused primarily on evaluating PIs originating from Japan, Vietnam and Indonesia after results from early tests revealed that most of the resistant accessions with resistance in the USA were from those regions (Walker et al. 2011, 2014b). In addition, most of the named *Rpp* genes were discovered in PIs from southern Japan (*Rpp1*, *Rpp2* and at least three alleles at the *Rpp5* locus), central Indonesia (*Rpp6*), or Vietnam (*Rpp7*). Although PI 462312 (‘Ankur’), the source of the *Rpp3* gene, was selected in Uttar Pradesh in north-central India, it was selected from a cross between unknown parents made in the USA, so the original source of the resistance gene is not known (Germplasm Resources Information Network). In this study, 84% of the accessions from Japan, 69% of those from Vietnam, and 49% of those from Indonesia had negative BLUP values, compared to only 11% of the 27 Chinese PIs tested. Nevertheless, some PIs from Japan, Vietnam or central Indonesia were susceptible in our tests; the Japanese accessions PI 200456, PI 200526 (Shiranui) and PI 224270 (Hougyoku) had positive BLUP values, even though Yamanaka et al. (2010) had reported that they were highly resistant to a bulk fungal isolate from central Japan. In contrast, PI 200487 was resistant in central Japan and also in our field tests.

PI 635999 (‘DT 2000’) and PI 423972 were also resistant in field studies conducted in Hanoi, Vietnam, in 2006 and 2007 (Pham et al. 2010). Some other accessions were susceptible in our tests but showed resistance to field populations of *P. pachyrhizi* in northern Vietnam. Among those were the *Rpp* differentials PI 230970 (*Rpp2*), which had an RB reaction in Hanoi, and PI 459025B (*Rpp4*), which had an intermediate reaction there. In contrast, PI 200492 (*Rpp1*), PI 462312 (*Rpp3*) and PI 417089A (allele at *Rpp3* locus) were resistant in our tests but developed TAN infection types in Hanoi (Pham et al. 2010). The reactions of some *Rpp* gene differentials in 2006 field tests in eastern Paraguay also differed from their reactions in our tests (Miles et al. 2008). PI 200492 and PI 615437, which has an *Rpp3* resistance allele, were both susceptible in Paraguay, whereas PI 230970 and some PI 567099A plants (with the recessive *rpp3* allele) were resistant there. These results attest to the pathogenic diversity among *P. pachyrhizi* populations.

Accessions from other countries that were resistant in our tests included PIs 368039, 379621 and 518295 from Taiwan, PI 423972 from Nepal, and PIs 203398, 417503 and 628932 from Brazil. PI 368039 was one of the few resistant PIs from this study that was also highly resistant to an unpurified rust isolate from central Japan (Yamanaka et al. 2010). The resistant accession PI 476897 is reported to be from China but was obtained from a germplasm collection in Hanoi, Vietnam. Harris et al. (2015) reported that PI 518295 has a resistance allele at the *Rpp1* locus, PI 417503 has one at the *Rpp3* locus, and PI 476905A has a resistance gene at the *Rpp6* locus. It was somewhat surprising that very few of the accessions from southern China were resistant in our tests, especially since at least seven of them have *Rpp* genes that are effective against foreign isolates and populations of the fungus (Supplemental Table S1). Six of 24 Chinese PIs screened are known or thought to have a resistance gene at the *Rpp1* locus, but like the *Rpp1-b* gene of PI 594538A and the *Rpp1* allele from PI 561356, none of their alleles provided resistance in the southern USA (Supplemental Table S1; Walker et al. 2011, 2014b). A few other Chinese accessions in our study had negative BLUP values that were only slightly less than zero, so they are unlikely to be of value as sources of SBR resistance genes.

A high percentage of soybean accessions with resistance in the USA have a resistance allele at the *Rpp3* locus (Harris et al. 2015), and many of them originated from southern Japan (Supplemental Table S1). At least 14 (28%) of the accessions with the 50 lowest BLUP values in the present study have a resistance allele at the *Rpp3* locus, so it is not surprising that a marker near this locus had the highest significance level in the GWAS (Fig. 3). Alleles at the *Rpp3* locus provided resistance to accessions from Indonesia (e.g., PI 567046A and PI 567034), Vietnam (PI 635999) and several from Japan, such as PI 416826A and PI 200488. Hyuuga and PI 462312 had similar BLUP means (− 0.64 and − 0.66), suggesting that the *Rpp5* allele in Hyuuga may not have enhanced resistance against US rust populations. Resistance genes at the *Rpp6* locus on Chr 18 appear to be less common, but the allele from PI 567102B and PI 567104B conditioned very high levels of resistance, resulting in the lowest and fourth lowest BLUP means. PI 567090, which had the third lowest BLUP value, has a resistance allele at the *Rpp3* locus and another on Chr 18, likely at the *Rpp6* locus (Harris et al. 2015).

The reactions of most of the *Rpp* gene differentials to field populations in this study differed considerably from the reactions of these accessions to a diverse collection of international and pre-2005 US isolates in a recent study by Stone et al. (2022). In that study, all 16 isolates defeated the *Rpp1* gene in PI 200492 to some extent, even though PI 200492 had one of the lowest BLUP values in the present study. In contrast, the *Rpp2* and *Rpp4* genes, which were ineffective in this study, conditioned resistance to all or most of the 14 isolates in the Stone et al. (2022) assays, as did the *Rpp1-b* gene from PI 594538A and the *Rpp1* alleles in three other accessions from China. PI 567102B (*Rpp6*) was the only highly resistant accession from the present study that also had resistance to most of the 16 isolates. These results and those of Pham et al. (2009) indicate a possible pathotype shift between the founder populations of *P. pachyrhizi* discovered in late 2004 and the predominant pathotypes of fungal populations along the Gulf Coast of the USA a few years later.

Detection of the genomic regions containing the *Rpp3* and *Rpp6* loci demonstrated that the GWAS methods used (i.e., the FarmCPU model and the FDR significance threshold) were effective. Of the eight genomic regions that were significant at *P* < 1.445 × 10^–5^; − log_10_(*P*) = 4.8, only ss715594707 on Chr 6 and ss715632525 on Chr 18 were locations with known *Rpp* loci. The former is near the *Rpp3* locus, a common location for genes that condition resistance to SBR in the USA (Supplemental Table S1; Harris et al. 2015), and the latter is near the *Rpp6* locus. Since at least four of the 20 PIs with the lowest BLUP values (ranging from − 1.457 to − 0.754) are known or thought to have an SBR resistance gene at the *Rpp3* locus (Harris et al. 2015; Vuong et al. 2016), it was not surprising that a marker near the locus was significantly associated with disease. In contrast, resistance alleles at the *Rpp6* locus have only been reported in a few PIs, but those alleles have conditioned much higher levels of resistance than any known *Rpp3* alleles. This was demonstrated by the low BLUP values of the Indonesian accessions PI 567102B and PI 567104b and the significance of the ss715632525 marker near the *Rpp6* locus on Chr 18. Since PI 567,090 also has the same base residue at that marker, it might also have a resistance gene at the *Rpp6* locus. The *Rpp6* gene is one of the few named genes that have been effective in both North and South America (Miles et al. 2008; Walker et al. 2014b). PIs 476905A, 567068A, 567076 and 567129 may also likely have a resistance gene at the *Rpp6* locus (Harris et al. 2015), but their BLUP values ranged from − 0.35 to − 0.56, suggesting that they carry a different allele from the one in PI 567102B and PI 567104B.

When Chang et al. (2016) performed a GWAS for SBR resistance using infection type data from the Miles et al. (2006) greenhouse assays, they found one significant marker near the *Rpp1* locus on Chr 18 and another on Chr 15. The *Rpp1* and *Rpp6* loci are both on Chr 18 but are located on different telomeres (Kim et al. 2012; Li et al. 2012; Yu et al. 2015). Although PI 200492 was one of the most resistant accessions in our study, we did not detect any significant markers close to the *Rpp1* locus. At least 11 of the PIs in the panel have a resistance allele at the *Rpp1* locus, but the allele(s) in seven Chinese accessions were ineffective against the US field pathotypes. As a result, the frequency of *Rpp1* alleles with significant phenotypic effects in this study was low, which probably explains the reason that the locus was not detected using GWAS. Other than PI 200492, PIs 417120 and 423958 from Japan, and PI 518295 from Taiwan were among the few accessions screened that had an effective resistance allele at the *Rpp1* locus (Supplemental Table S1).

The failure of the GWAS analysis to detect significant markers close to *Rpp2* on Chr 16; (Yu et al. 2015), *Rpp4* at the opposite end of Chr 18 from *Rpp1* and *Rpp6* (Silva et al. 2008), *Rpp5* on Chr 3 (Garcia et al. 2008), or *Rpp7* on Chr 19 (Childs et al. 2018b) was likely due to a low frequency of resistance genes and/or weak phenotypic effects. Although PI 605823 (*Rpp7*) and PI 200487 (allele at *Rpp5*) were among the 20 accessions with the lowest BLUP means for disease in this study, *Rpp7* has not been reported in any other accessions, and PI 471904 appears to be the only other PI with an effective allele at the *Rpp5* locus. A failure to detect genes that are present at a low frequency in a population is one limitation of GWAS analyses (Bandillo et al. 2015).

The detection of significant markers in regions of six chromosomes that have not been reported to have *Rpp* loci was unexpected, and the fact that the marker significance levels were higher than that of the marker close to the *Rpp6* locus is encouraging. Some of the resistant accessions that we tested may have novel resistance genes in those regions that could be used in *Rpp* gene pyramids to improve resistance. Because a majority of the PIs in the GWAS panel had been reported by Miles et al. (2006) to be susceptible or to have had mixed reactions to SBR, few of them have been used to develop biparental mapping populations. It is possible that the SBR disease rating data that we used for the GWAS may have allowed contributions of *Rpp* loci in the six regions to be detected for the first time. Much of the phenotypic data used the BLUP values used for GWAS in this study reflected both disease severity and intensity of urediniospore production. Those semi-quantitative data provided a more accurate assessment of reactions that were intermediate between heavily sporulating TAN infection types and Type 0 or RB infection types with few uredinia and low sporulation. Yamanaka et al. (2010) also recognized the value of using semi-quantitative criteria (i.e., rating scales) for more accurate assessments of host reactions to SBR. Although Chang et al. (2016) also reported a novel putative resistance QTL between positions 10,659,000 and 10,859,000 bp on Chr 15, it did not correspond to the genomic region that we detected near position 15,694,109 on that chromosome.

Findings from this study should be useful for the development of cultivars with broader and more durable SBR resistance. Some accessions previously reported be susceptible to SBR should be re-evaluated for resistance in some other countries where rust is an economically important disease. Confirmation of an unknown *Rpp* locus in any of the six previously unreported genomic regions that were detected by GWAS would offer soybean breeders additional options for developing cultivars with novel combinations of resistance genes. Data collected early in this study led to the discovery of the *Rpp7* locus in PI 605823 (Childs et al. 2018b). After the resistant accessions responsible for the significant GWAS markers are identified, their resistance can be characterized using a panel of pathogenically diverse *P. pachyrhizi* isolates, and biparental mapping populations can be created to fine-map the locations of the novel loci.

## Supplementary Information

Below is the link to the electronic supplementary material.

Supplementary file1 (DOCX 58 kb)

## Data Availability

Supplementary Table S1 contains the BLUP values for all accessions used for the GWAS analysis. The disease rating data for PIs grown in each year-location environment are available from the corresponding authors on reasonable request.
