# Supplementary material for: Genomic regions associated with resistance to soybean rust (Phakopsora pachyrhizi) under field conditions in soybean germplasm accessions from Japan, Indonesia and Vietnam
Source: Theor Appl Genet. 2022 Jul 28;135(9):3073–86. doi: 10.1007/s00122-022-04168-y (PMC9482582; doi:10.1007/s00122-022-04168-y)
Supplement: Supplementary file 1 — Supplementary file1 (DOCX 58 kb) [file 122_2022_4168_MOESM1_ESM.docx]

**Supplemental Table S1**. Susceptible checks, *Rpp* differentials and germplasm accessions evaluated for their reactions to soybean rust infection, which are summarized as best linear unbiased predictor (BLUP) values.

|  |  |  | **Miles et al.** |  |  |  |  |
| --- | --- | --- | --- | --- | --- | --- | --- |
|  | **BLUP** | ***Rpp* gene(s)** | **(2006)** |  |  |  |  |
| **Accession** | **value^a^** | **[unconfirmed]^b^** | **reaction^c^** | **Name** | **MG** | **State or locality** | **Country (region)** |
| PI 586981 | 0.85 | none (susceptible check) | S | KS4694 | IV | Kansas | United States (central) |
| PI 518664 | 0.98 | none (susceptible check) | S | Hutcheson | V | Virginia | Unted States (southeastern) |
| PI 615582 | 1.40 | none (susceptible check) | S | Caviness | V | Arkansas | United States (south-central) |
| PI 630984 | 1.00 | none (susceptible check) | S | 5601T | V | Tennessee | United States (southeastern) |
| PI 511813 | 1.20 | none (susceptible check) | S | Twiggs | VI | Georgia | United States (southeastern) |
| PI 548986 | 0.85 | none (susceptible check) | S | Brim | VI | North Carolina | United States (southeastern) |
| PI 599333 | 1.07 | none (susceptible check) | S | Musen | VI | South Carolina | United States (southeastern) |
| PI 617045 | 0.53 | none (susceptible check) | S | NC-Roy | VI | North Carolina | United States (southeastern) |
| PI 584506 | 1.14 | none (susceptible check) | S | Carver | VII | Alabama | United States (southeastern) |
| PI 595645 | 1.07 | none (susceptible check) | S | Benning | VII | Georgia | United States (southeastern) |
| PI 641156 | 1.27 | none (susceptible check) | S | NC-Raleigh | VII | North Carolina | United States (southeastern) |
| PI 568236 | 1.36 | none (susceptible check) | S | Maxcy | VIII | South Carolina | United States (southeastern) |
| PI 608033 | 1.38 | none (susceptible check) | S | Kuell | VIII | Alabama | United States (southeastern) |
| PI 612157 | 1.60 | none (susceptible check) | S | Prichard | VIII | Georgia | United States (southeastern) |
| PI 548969 | 0.79 | none (susceptible check) | S | Alamo | IX | Florida | United States (southeastern) |
| PI 556805 | 1.12 | none (susceptible check) | S | H9190 | IX | unknown | United States (southeastern) |
| PI 200492 | -1.33 | *Rpp1* | Mixed | Komata | VII | Shikoku | Japan (southern) |
| PI 561356 | 0.92 | allele at *Rpp1* | Mixed | Jin yun dou | V | Jiangsu | China (east-central) |
| PI 594538A | 0.99 | *Rpp1-b* | Mixed | Min hou bai sha wan dou | IX | Fujian | China (southeastern) |
| PI 230970 | 0.12 | *Rpp2* | Mixed |  | VII | unknown | Japan (unknown) |
| PI 224270 | 0.12 | *rpp2* | S | Hougyoku | VII | Hyogo | Japan (southern) |
| PI 462312 | -0.66 | *Rpp3* | S | Ankur | VIII | Uttar Pradesh/Florida | India/United States |
| PI 459025B | 0.12 | *Rpp4* | Mixed | (Bing nan) | VIII | Fujian | China (southeastern) |
| PI 200456 | 0.97 | *rpp5* | S | Awashima Zairai | VIII | Shikoku | Japan (southern) |
| PI 200487 | -0.80 | allele at *Rpp5* | S | Kinoshita | VIII | Shikoku | Japan (southern) |
| PI 200526 | 1.24 | allele at *Rpp5* | S | Shiranui | VIII | Shikoku | Japan (southern) |
| PI 471904 | -0.61 | allele at *Rpp5* | S | Orba | IX | Java | Indonesia (central) |
| PI 567102B | -1.16 | *Rpp6* | R |  | IX | East Java | Indonesia |
| PI 567104B | -1.45 | allele at *Rpp6* | S |  | IX | East Java | Indonesia |
| PI 605823 | -0.97 | *Rpp7* | Mixed |  | IX | Ha Giang | Vietnam (northern) |
| PI 506764 | -0.64 | *Rpp3* + *Rpp5* allele | S | Hyuuga | VII | Kyūshū | Japan (southern) |
| FC 31745 | -0.07 |  | S |  | VI |  | unknown |
| PI 90499 | 0.95 |  | S | Black and white | VI | Hebei | China (northeastern) |
| PI 97081 | -0.07 |  | S |  | VI | Hwanghae Puk | Korea (northern) |
| PI 165943 | 0.41 |  | S | Bhart | VII | Uttar Pradesh | India (north-central) |
| PI 166438 | 0.63 |  | S |  | X | unknown | India |
| PI 174852 | 0.76 |  | S | Bhart | IX | Uttar Pradesh | India (north-central) |
| PI 197182 | -0.06 | [alleles at *Rpp2* & *Rpp3*] | S | Raub 16.1422 | VIII | unknown | Malaysia |
| PI 200445 | -0.16 | [allele at *Rpp3*] | S | Aka Nida | VIII | Shikoku | Japan (southern) |
| PI 200455 | -0.47 |  | Mixed | Aso No. 1 | VIII | Shikoku | Japan (southern) |
| PI 200461 | -0.59 |  | S | Chiya Kotsubu | VI | Shikoku | Japan (southern) |
| PI 200464 | -0.69 |  | S | Daizu Uchida | VII | Shikoku | Japan (southern) |
| PI 200465 | -0.18 |  | S | Fusanari Daizu | VIII | Shikoku | Japan (southern) |
| PI 200466 | -0.55 |  | Mixed | Gaku Bun | VII | Shikoku | Japan (southern) |
| PI 200476 | -0.16 |  | S | Hito Yoshi | VII | Shikoku | Japan (southern) |
| PI 200477 | -0.44 |  | S | Hondo Daizu | VII | Shikoku | Japan (southern) |
| PI 200488 | -0.70 | [allele at *Rpp3*] | Mixed | Kiro Aki Daizu | VIII | Shikoku | Japan (southern) |
| PI 200490 | -0.48 |  | S | Kiwami | VIII | Shikoku | Japan (southern) |
| PI 200502 | -0.72 |  | S | Misao | VI | Shikoku | Japan (southern) |
| PI 200505 | -0.18 |  | S | Nagahashi | VI | Shikoku | Japan (southern) |
| PI 200507 | -0.35 |  | S | Natsu Daizu No. 1 | VIII | Shikoku | Japan (southern) |
| PI 200509 | -0.73 |  | S | Nishimura Daizu | VIII | Shikoku | Japan (southern) |
| PI 200515 | -0.48 |  | S | Oku Kuro Daizu | VIII | Shikoku | Japan (southern) |
| PI 200520 | -0.10 |  | S | Ono | VIII | Shikoku | Japan (southern) |
| PI 200521 | -0.44 |  | S | Oura | IX | Shikoku | Japan (southern) |
| PI 200523 | -0.55 |  | S | San Goku | VIII | Shikoku | Japan (southern) |
| PI 200524 | -0.75 |  | S | Shimo Baba | VIII | Shikoku | Japan (southern) |
| PI 200525 | -0.52 |  | Mixed | Shimatsu Ura | VIII | Shikoku | Japan (southern) |
| PI 200528 | 0.96 |  | S | Shiro Daizu | VIII | Shikoku | Japan (southern) |
| PI 200531 | -0.41 |  | S | Shiro Daizu | VIII | Shikoku | Japan (southern) |
| PI 200532 | -1.13 |  | S | Shiro Hanasaki No. 1 | VIII | Shikoku | Japan (southern) |
| PI 200539 | -0.02 |  | S | Suzanari | VII | Shikoku | Japan (southern) |
| PI 200544 | 0.19 |  | S | Tanba Kuro | VII | Shikoku | Japan (southern) |
| PI 200547 | -0.92 |  | S | Waka Shima | VIII | Shikoku | Japan (southern) |
| PI 200551 | -0.52 |  | S | Yonekadake | VIII | Shikoku | Japan (southern) |
| PI 203398 | -0.23 |  | S | Abura | VIII | unknown | Brazil |
| PI 238109 | 0.15 |  | R | Jugatsu Shiromame | X | Okinawa | Japan (southern) |
| PI 281890D | 0.35 |  | S |  | IX | unknown | Indonesia |
| PI 281891A | 0.63 |  | S |  | IX | unknown | Indonesia |
| PI 281898A | 0.71 |  | S |  | IX | unknown | Indonesia |
| PI 281905 | 0.71 |  | S |  | IX | unknown | Malaysia |
| PI 307868 | 0.56 |  | S |  | X | Madhya Pradesh | India (central) |
| PI 307873B | 0.43 |  | S |  | IX | Madhya Pradesh | India (central) |
| PI 307880B | 0.45 |  | S |  | IX | Madhya Pradesh | India (central) |
| PI 307880C | -0.13 |  | S |  | X | Madhya Pradesh | India (central) |
| PI 307882C | 0.50 |  | S |  | X | Madhya Pradesh | India (central) |
| PI 309655B | 0.63 |  | S |  | IX | unknown | Malaysia |
| PI 309655D | 0.76 |  | S |  | IX | unknown | Malaysia |
| PI 323567 | 0.71 |  | S |  | IX | Uttar Pradesh | India (north-central) |
| PI 323577 | 0.50 |  | S |  | IX | Uttar Pradesh | India (north-central) |
| PI 341244A | 0.53 |  | S | Yellow Kedele | IX | unknown | Tanzania |
| PI 341248 | 0.76 |  | S | Sangalo | IX | unknown | Tanzania |
| PI 341249 | 0.48 |  | S |  | IX | unknown | Tanzania |
| PI 341258 | 0.71 |  | S | HLS 154 | IX | unknown | Tanzania |
| PI 342003 | 0.20 |  | S | Hankuho | V | unknown | Japan |
| PI 368039 | -0.61 |  | S | Tainung No. 4 | VI | unknown | Taiwan |
| PI 371607 | 0.27 |  | S | Red China PB 1 | VI | unknown | Pakistan |
| PI 379621 | -0.21 |  | Mixed | R10 | VI | unkown | Taiwan |
| PI 398458 | 0.27 |  | S |  | V | Kangwon | Korea (southern) |
| PI 398734 | 0.34 |  | S |  | VI | Kangwon | Korea (southern) |
| PI 407767 | 0.63 |  | S |  | IX | Guangdong | China (east-central) |
| PI 408059 | 0.27 |  | S |  | V | Kyongsang Puk | Korea (southern) |
| PI 416778 | -0.18 |  | R | Aki Sengoku (Kyushu 11) | VIII | Kyūshū | Japan (southern) |
| PI 416806 | -0.83 |  | S | Aso Aogari (Kyushu 27) | VIII | Kyūshū | Japan (southern) |
| PI 416810 | -0.55 | [allele at *Rpp3*] | S | Ban Kuru Daizu | IX | Kyūshū | Japan (southern) |
| PI 416825A | 0.50 |  | S | Cha Sengoku 81 | IX | Kinki | Japan (southern/central) |
| PI 416826A | -0.78 | [allele at *Rpp3*] | Mixed | Cha Sengoku 81 | VIII | unknown | Japan |
| PI 416873A | -0.51 |  | S | Fusanari daizu | VIII | Kyūshū | Japan (southern) |
| PI 416873B | -0.20 | [allele at *Rpp3*] | Mixed | (Fusanari daizu) | VIII | Kyūshū | Japan (southern) |
| PI 416874A | -0.55 |  | S | Fusanari 1 | IX | Kyūshū | Japan (southern) |
| PI 416880A | -0.07 |  | S | Gifu wase senshutsu | IX | Kinki | Japan (south-central) |
| PI 416886 | -0.16 | [allele at *Rpp3*] | Mixed | Ginsui Zairai | VIII | Kyūshū | Japan (southern) |
| PI 416893 | -0.01 |  | S | Hachigatsu Daizu | VII | Shikoku | Japan (southern) |
| PI 416935 | -0.41 |  | S | Hoshino Zairai | VIII | Kyūshū | Japan (southern) |
| PI 416961 | 0.07 |  | S | Izari 96 | VIII | Kinki | Japan (south-central) |
| PI 417013 | -0.27 | [allele at *Rpp3*] | Mixed | Kawahara | VIII | Kyūshū | Japan (southern) |
| PI 417014A | -0.44 |  | S | Kawahara 1 | IX | Kyūshū | Japan (southern) |
| PI 417085 | -0.78 | [allele at *Rpp3*] | S | Kumaji 1 | IX | Kyūshū | Japan (southern) |
| PI 417089A | -0.65 | [allele at *Rpp3*] | R | Kuro daizu | IX | Kyūshū | Japan (southern) |
| PI 417089B | -0.61 | [allele at *Rpp3*] | R | (Kuro daizu) | IX | Kyūshū | Japan (southern) |
| PI 417116 | -0.33 | [allele at *Rpp3*] | Mixed | Kyushu 19 | VII | Kyūshū | Japan (southern) |
| PI 417119 | -0.48 | [allele at *Rpp3*] | S | Kyushu 24 | VIII | Kyūshū | Japan (southern) |
| PI 417120 | -0.65 | [allele at *Rpp1*] | Mixed | Kyushu 25 | VIII | Kyūshū | Japan (southern) |
| PI 417125 | -0.54 | allele at *Rpp2* | R | Kyushu 31 | VIII | Kyūshū | Japan (southern) |
| PI 417126 | -0.08 |  | R | Kyushu 32 | VIII | Kyūshū | Japan (southern) |
| PI 417128 | -0.59 | [allele at *Rpp3*] | Mixed | Kyushu 37 | VII | Kyūshū | Japan (southern) |
| PI 417129B | -0.68 | [>1 *Rpp* gene] | S | (Kyushu 40) | IX | Kyūshū | Japan (southern) |
| PI 417132 | -0.32 | [allele at *Rpp3*] | Mixed | Kyushu 56 | VII | Kyūshū | Japan (southern) |
| PI 417134 | -0.25 |  | Mixed | Magarikawa Zairai | VIII | Kyūshū and Okinawa | Japan (southern) |
| PI 417208 | -0.38 |  | Mixed | Oka Kaizu | VIII | Kyūshū and Okinawa | Japan (southern) |
| PI 417335 | -0.07 |  | S | Shirosota | V | Kanto | Japan (central) |
| PI 417503 | -0.52 | [allele at *Rpp3*] | Mixed | Pioneira | VI | unknown | Brazil |
| PI 423957 | -0.62 | allele at *Rpp5* | S | Ano 2 | VIII | Kumamoto | Japan (southern) |
| PI 423958 | -0.54 | [allele at *Rpp1*] | S | Asoaogari | VIII | Kumamoto | Japan (southern) |
| PI 423959 | -0.83 | [probably two genes] | S | Asomusume | VIII | Kumamoto | Japan (southern) |
| PI 423960A | -0.47 | [>1 *Rpp* gene] | S | Gokudaizu | IX | Kumamoto | Japan (southern) |
| PI 423960B | 0.05 |  | S | (Gokudaizu) | IX | Kumamoto | Japan (southern) |
| PI 423961A | -0.19 | [allele at *Rpp3*] | S | Hondo | IX | Kumamoto | Japan (southern) |
| PI 423961B | 0.71 |  | S | (Hondo) | IX | Kumamoto | Japan (southern) |
| PI 423962 | -0.36 | [allele at *Rpp3*] | S | Hyuga | VIII | Kumamoto | Japan (southern) |
| PI 423963 | -0.58 |  | S | Izumi | VIII | Kumamoto | Japan (southern) |
| PI 423966 | -0.52 | [allele at *Rpp3*] | S | Kumaji 2 | VIII | Kumamoto | Japan (southern) |
| PI 423968 | 1.29 |  | S | Oita Akidaizu 1 | VIII | Kumamoto | Japan (southern) |
| PI 423971C | 0.64 |  | S | (Ozu ura) | VIII | Kumamoto | Japan (southern) |
| PI 423972 | -0.68 | [allele at *Rpp4*] | R | Takema | IX | Kumamoto | Japan (southern) |
| PI 438439 | -0.27 |  | S | VIR 5775 | VII | unknown | Nepal |
| PI 441373B | 0.51 |  | S | (Hitam) | VIII | Java | Indonesia (south-central) |
| PI 458278B | -0.01 |  | S | KAS 580-4 | V | Cholla Nam | Korea (southern) |
| PI 459025A | 0.86 |  | S | Bing nan | IX | Fujian | China (southeastern) |
| PI 468374B | 0.63 |  | S | (Manipur) | IX | Manipur | India (northeast) |
| PI 476882 | 0.62 |  | S | Ba-Vi | VII | Northern Vietnam | Vietnam (northern) |
| PI 476889 | 0.48 |  | S | Den Bac Ha | V | Northern Vietnam | Vietnam (northern) |
| PI 476897 | -0.37 |  | S | Hoa-an | VI | Northern Vietnam | Vietnam (northern) |
| PI 476905A | -0.56 | [allele at *Rpp6*] | Mixed | Nguu mao hong | V | unknown | China |
| PI 476920 | 0.69 |  | S | Tung nghia 2 | V | Southern Vietnam | Vietnam (southern) |
| PI 497952 | 0.71 |  | S | I.C. 191 | X | Bihar | India (eastern) |
| PI 497964B | 0.76 |  | S | (I.C. 9461) | IX | Sikkim | India (northeastern) |
| PI 506491 | -0.08 | [allele at *Rpp3*] | S | Akanida | VIII | Kyūshū | Japan (southern) |
| PI 506504 | 0.88 |  | S | Aki Daizu | VII | Kanto | Japan (central) |
| PI 506664 | 0.20 |  | S | Dekisugi 1 | VI | Kinki | Japan (southern/central) |
| PI 506677 | 0.54 |  | Mixed | Fusanari Daizu | VIII | Kyūshū | Japan (southern) |
| PI 506695 | -0.07 | [allele at *Rpp3*] | S | Gogaku | VI | Kyūshū | Japan (southern) |
| PI 506938 | -0.75 |  | S | Koukei 86 | VI | Kyūshū | Japan (southern) |
| PI 506939 | -0.36 |  | S | Koukei 202 | VI | Kyūshū | Japan (southern) |
| PI 506947 | -0.43 | [allele at *Rpp3*] | Mixed | Kumaji 2 | VIII | Kyūshū | Japan (southern) |
| PI 507004 | -0.36 | [allele at *Rpp3*] | S | Kyuushuu 33 | VIII | Kyūshū | Japan (southern) |
| PI 507005 | -0.21 | [allele at *Rpp3*] | S | Kyuushuu 35 | VII | Kyūshū | Japan (southern) |
| PI 507007 | -0.04 |  | S | Kyuushuu 39 | VI | Kyūshū | Japan (southern) |
| PI 507008 | -0.21 | [allele at *Rpp3*] | S | Kyuushuu 41 | VII | Kyūshū | Japan (southern) |
| PI 507009 | -0.25 | [allele at *Rpp3*] | S | Kyuushuu 43 | VI | Kyūshū | Japan (southern) |
| PI 507023 | 0.71 |  | S | Manshuu Kuro Sengoku | VIII | Kyūshū | Japan (southern) |
| PI 507035 | 0.88 |  | S | Menka Daizu | VIII | Shikoku | Japan (southern) |
| PI 507259 | 0.23 | [allele at *Rpp3*] | R | Shrio Daizu | VII | Kyūshū | Japan (southern) |
| PI 518295 | -0.46 | [allele at *Rpp1*] | S | Kao Hsiung 8 | VII | unknown | Taiwan |
| PI 549020 | 0.27 |  | S | Lu cha dou | V | Liaoning | China (northeastern) |
| PI 566956 | -0.51 |  | S | MARIF 2502 | IX | East Java | Indonesia (central) |
| PI 566957 | -0.32 |  | S | MARIF 2503 | IX | East Java | Indonesia (central) |
| PI 566961 | 0.29 |  | S | MARIF 2507 | VIII | East Java | Indonesia (south-central) |
| PI 566963 | -0.53 |  | S | MARIF 2509 | IX | East Java | Indonesia (central) |
| PI 566964A | 0.04 |  | R | MARIF 2510 | IX | East Java | Indonesia (central) |
| PI 566970A | 0.09 |  | S | MARIF 2516 | IX | East Java | Indonesia (central) |
| PI 566974 | -0.68 |  | S | MARIF 2520 | IX | East Java | Indonesia (central) |
| PI 566975 | -0.84 |  | S | MARIF 2521 | VIII | East Java | Indonesia (central) |
| PI 566979B | 0.20 |  | S | (MARIF 2525) | IX | East Java | Indonesia (central) |
| PI 566982 | -0.79 |  | S | MARIF 2528 | IX | East Java | Indonesia (central) |
| PI 566984 | -0.89 |  | S | MARIF 2532 | VI | East Java | Indonesia (central) |
| PI 566987A | 0.06 |  | S | MARIF 2536 | VIII | East Java | Indonesia (central) |
| PI 566988A | -0.58 |  | S | MARIF 2537 | VIII | East Java | Indonesia (central) |
| PI 567009B | 0.71 |  | S | (MARIF 2558) | IX | East Java | Indonesia (central) |
| PI 567018 | 0.76 |  | S | MARIF 2585 | IX | East Java | Indonesia (central) |
| PI 567020A | -0.63 | [allele at *Rpp3*] | S | MARIF 2587 | VIII | unknown | Indonesia (central) |
| PI 567024 | -0.52 | [allele at *Rpp3*] | R | MARIF 2591 | VIII | unknown | Indonesia (central) |
| PI 567025A | -0.75 | [allele at *Rpp3*] | Mixed | MARIF 2592 | VIII | unknown | Indonesia (central) |
| PI 567031B | 0.59 | [>1 *Rpp* gene] | Mixed | (MARIF 2604) | VIII | Central Java | Indonesia (central) |
| PI 567034 | -0.81 | [allele at *Rpp3*] | Mixed | MARIF 2607 | VIII | Central Java | Indonesia (central) |
| PI 567039 | -0.63 | [allele at *Rpp3*] | S | MARIF 2618 | VII | East Java | Indonesia (central) |
| PI 567046A | -0.83 | [allele at *Rpp3*] | R | MARIF 2627 | VIII | Central Java | Indonesia (central) |
| PI 567047B | 0.63 |  | S | (MARIF 2628) | IX | East Java | Indonesia (central) |
| PI 567049A | 0.75 |  | S | MARIF 2631 | VIII | East Java | Indonesia (central) |
| PI 567052 | 0.26 |  | S | MARIF 2634 | IX | East Java | Indonesia (central) |
| PI 567053 | -0.43 | [allele at *Rpp3*] | Mixed | MARIF 2635 | IX | East Java | Indonesia (central) |
| PI 567054C | -0.51 | [allele at *Rpp3*] | S | (MARIF 2647) | IX | East Java | Indonesia (central) |
| PI 567055 | 0.37 |  | S | MARIF 2648 | VIII | East Java | Indonesia (central) |
| PI 567056A | -0.66 | [allele at *Rpp3*] | R | MARIF 2649 | VIII | unknown | Indonesia (central) |
| PI 567058D | -0.47 | [allele at *Rpp3*] | Mixed | (MARIF 2651) | IX | unknown | Indonesia (central) |
| PI 567059 | -0.46 | [undetermined] | Mixed | MARIF 2654 | V | East Java | Indonesia (central) |
| PI 567061 | -1.09 | [probably two genes] | S | MARIF 2657 | VIII | East Java | Indonesia (central) |
| PI 567067B | 0.71 |  | S | (MARIF 2665) | IX | East Java | Indonesia (central) |
| PI 567068A | -0.47 | [allele at *Rpp6*] | S | MARIF 2666 | VII | East Java | Indonesia (central) |
| PI 567069A | 0.24 |  | S | MARIF 2667 | VIII | East Java | Indonesia (central) |
| PI 567076 | -0.35 | [allele at *Rpp6*] | S | MARIF 2674 | VII | East Java | Indonesia (central) |
| PI 567079 | 0.45 |  | S | MARIF 2677 | VIII | East Java | Indonesia (central) |
| PI 567090 | -1.24 | [*Rpp3* + *Rpp6* (probably)] | S | MARIF 2688 | IX | East Java | Indonesia (central) |
| PI 567099A | 0.83 | *rpp3* | R | MARIF 2740 | IX | East Java | Indonesia (central) |
| PI 567123A | -0.37 | [> 1 *Rpp* gene] | Mixed | MARIF 2790 | VIII | East Java | Indonesia (central) |
| PI 567123B | 0.51 |  | S | (MARIF 2790) | IX | East Java | Indonesia (central) |
| PI 567129 | -0.48 | [allele at *Rpp6*] | Mixed | MARIF 2796 | IX | East Java | Indonesia (central) |
| PI 567135A | 0.43 |  | S | MARIF 2802 | IX | East Java | Indonesia (central) |
| PI 567142 | 0.76 |  | S | MARIF 2813 | IX | Bali | Indonesia (central) |
| PI 567143 | 0.71 |  | S | MARIF 2814 | IX | Bali | Indonesia (central) |
| PI 567146B | 0.76 |  | S | (MARIF 2817) | IX | Bali | Indonesia (central) |
| PI 567147A | 0.38 |  | S | MARIF 2818 | IX | Bali | Indonesia (central) |
| PI 567180 | -0.08 | [allele at *Rpp3*] | S | 140 | V | unknown | Vietnam |
| PI 567188 | -0.66 |  | S | VX 9-3 | VI | unknown | Vietnam |
| PI 567189A | -0.69 |  | Mixed | Ekhabac | IV | unknown | Vietnam |
| PI 567190 | -0.45 | [undetermined] | Mixed | Halang 4 thang | VI | unknown | Vietnam |
| PI 567191 | -0.12 |  | S | Ouesso | V | unknown | Vietnam |
| PI 567634 | 0.20 |  | S | Mi yang niu mao huang | V | Henan | China (central) |
| PI 578361 | 0.25 |  | S | Yi chang hei huang | X | unknown | China |
| PI 578457A | -0.40 | [allele at *Rpp3*] | Mixed | May Den | VIII | An Giang | Vietnam (southern) |
| PI 587609A | 0.50 |  | S | Qi dong xian hao dou No. 1 | IX | Jiangsu | China (east-central) |
| PI 587609B | 0.40 |  | S | (Qi dong xian hao dou No. 1) | IX | Jiangsu | China (east-central) |
| PI 587684A | 0.44 |  | S | Ai jiao huang | VI | Anhui | China (eastern) |
| PI 587741 | 0.74 |  | S | An lu niu mao huang | VII | Hubei | China (central) |
| PI 587880A | 0.50 | Unnamed allele at *Rpp1* | R | Huang dou | VI | Zhejiang | China (east-central) |
| PI 587855 | 0.86 | Allele at *Rpp1* | R | Jia Bai Jia | VIII | Zhejiang | China (east-central) |
| PI 587886 | 0.67 | Unnamed allele at *Rpp1* | R | Bai dou | VI | Zhejiang | China (east-central) |
| PI 587905 | 0.58 | Unnamed allele at *Rpp1* | R | Xiao Huang Dou | VII | Zhejiang | China (east-central) |
| PI 587939 | 0.33 |  | S | Tai ning huang pi dou | X | Fujian | China (southeastern) |
| PI 587948 | 0.61 |  | S | Zhao an qiu da dou | IX | Fujian | China (southeastern) |
| PI 587952 | 0.12 |  | S | Nin hua wu dou | X | Fujian | China (southeastern) |
| PI 594149 | -0.27 | [allele at *Rpp3*] | Mixed | Aso musume | VIII | Kumamoto, Kyūshū | Japan (southern) |
| PI 594172A | -0.26 | [allele at *Rpp3*] | R | Gogaku | VII | Kumamoto, Kyūshū | Japan (southern) |
| PI 594542 | 0.71 |  | S | Ming xi ai jiao hong hua dou | IX | Fujian | China (southeastern) |
| PI 594563 | 0.71 |  | S | Ai jiao tian dou | IX | Jiangxi | China (southeastern) |
| PI 594566 | 0.35 |  | S | Ji an huang dou | IX | Jiangxi | China (southeastern) |
| PI 594742 | -0.01 |  | S | Hua huang dou | IX | Guangxi | China (south-central) |
| PI 594755 | 0.68 |  | S | Liu yue huang dou | IX | Guangxi | China (south-central) |
| PI 594760B | 0.77 | *Rpp1-b* or similar allele | R | (Gou jiao huang dou) | IX | Guangxi | China (south-central) |
| PI 594767A | 0.51 | Allele at *Rpp1* | R | Zhao ping hei dou | IX | Guangxi | China (south-central) |
| PI 594772A | 0.07 |  | S | Pu bei tai wei da hei dou | IX | Guangxi | China (south-central) |
| PI 594796 | -0.07 | [recessive gene?] | Mixed | Xi bai dou | VIII | Yunnan | China (south-central) |
| PI 594848 | 0.68 |  | S | Xiao hei dou | IX | Yunnan | China (south-central) |
| PI 603584 | 0.34 |  | S | Qing dou | V | Shanxi | China (central) |
| PI 605770 | 0.47 |  | S | "Sample 33" | IV | Lang Son | Vietnam (northeastern) |
| PI 605773 | -0.24 | [undetermined] | Mixed | "Sample 36" | V | Cao Bang | Vietnam (northeastern) |
| PI 605774 | -0.27 | [allele at *Rpp3*] | S | "Sample 37" | V | Cao Bang | Vietnam (northeastern) |
| PI 605778A | 0.02 |  | S | "Sample 41" | VIII | Cao Bang | Vietnam (northeastern) |
| PI 605791A | -0.41 | [allele at *Rpp4*] | Mixed |  | VI | Cao Bang | Vietnam (northeastern) |
| PI 605823 | -0.98 |  | Mixed |  | IX | Ha Giang | Vietnam (north-central) |
| PI 605824A | -0.54 | [allele at *Rpp3*] | S | "Sample 88" | V | Ha Giang | Vietnam (north-central) |
| PI 605829 | -0.63 | [undetermined] | Mixed | "Sample 94" | V | Ha Giang | Vietnam (north-central) |
| PI 605833 | 0.16 |  | R | "Sample 102" | IX | Ha Giang | Vietnam (north-central) |
| PI 605838 | -0.36 | [allele at *Rpp3*] | Mixed | Xhanh si man | V | Ha Giang | Vietnam (north-central) |
| PI 605854B | -0.38 | [allele at *Rpp3*] | Mixed |  | V | Tuyen quang | Vietnam (north-central) |
| PI 605865B | -0.46 | [allele at *Rpp3*] | Mixed |  | V | Lao cai | Vietnam (north-central) |
| PI 605885B | -0.61 | [allele at *Rpp3*] | Mixed |  | V | Lao cai | Vietnam (north-central) |
| PI 605891A | -0.31 | [undetermined] | Mixed |  | V | Son La | Vietnam (northern) |
| PI 605891B | -0.53 | [allele at *Rpp3*] | Mixed |  | VI | Son La | Vietnam (northern) |
| PI 606397B | -0.36 | [allele at *Rpp3*] | Mixed | (Hat nho duc trong) | V | (northern) | Vietnam (northern) |
| PI 606405 | -0.61 | [allele at *Rpp3*] | Mixed | Madrak | IV | (northern) | Vietnam (northern) |
| PI 606440A | -0.71 |  | Mixed | VX 92 | IV | (northern) | Vietnam (northern) |
| PI 615437 | -0.64 | [allele at *Rpp3*] | S | A 9 | VI | (northern) | Vietnam (northern) |
| PI 615445 | -0.44 | [allele at *Rpp3*] | Mixed | Hi long 3 | VI | unknown | Vietnam (unknown) |
| PI 615483 | 0.76 |  | S | T 87 | VI | unknown | Vietnam (unknown) |
| PI 615484 | 0.60 |  | S | MTD 176 | VI | Can Tho | Vietnam (southern) |
| PI 615487 | 0.88 |  | S | Xanh tien dai | V | unknown | Vietnam (unknown) |
| PI 615488 | -0.43 |  | S | Ha lang | V | unknown | Vietnam (unknown) |
| PI 615498 | -0.38 |  | S | Vang muong khuong | VII | unknown | Vietnam (unknown) |
| PI 615501 | 0.63 |  | S | Bach hoa vang | VI | unknown | Vietnam (unknown) |
| PI 615508 | 0.07 |  | S | Ban doc a hat den | VI | unknown | Vietnam (unknown) |
| PI 628932 | -0.35 |  | S | FT-2 | VII | Paraná | Brazil (southern) |
| PI 632637 | -0.55 |  | S | IS 137 | V |  | Vietnam |
| PI 632639 B | -0.49 |  | S | (Hoang mao) | IV |  | Vietnam |
| PI 632641 B | -0.43 |  | S | (VG 384) | V |  | Vietnam |
| PI 632642 | -0.42 |  | S | DN 42 | IV |  | Vietnam |
| PI 632643 | 0.96 |  | S | M 103 | V |  | Vietnam |
| PI 632650 | -0.44 |  | S | DT 22 | VI |  | Vietnam |
| PI 632654 | -0.36 |  | S | VG 4763 | V |  | Vietnam |
| PI 632658 | -0.51 |  | S | V 49 | V |  | Vietnam |
| PI 632663 B | -0.63 |  | S | (H 5) | V |  | Vietnam |
| PI 632668 | -0.35 |  | S | H 10 | VI |  | Vietnam |
| PI 632944 B | -0.55 |  | S | (TN 12) | VI | Trung Quoc | Vietnam |
| PI 632945 A | -0.35 |  | S | Chu se | V | Gia Lai | Vietnam (central) |
| PI 635998 | 0.51 |  | S | DT80 | VII |  | Vietnam |
| PI 635999 | -1.09 |  | S | DT 2000 | VI |  | Vietnam |
| PI 636000 | 0.33 |  | S | Cuc Vang | IV |  | Vietnam |

^a^ Lower BLUP values indicate fewer soybean rust symptoms and a higher level of resistance in the field tests

^b^ Known and suspected *Rpp* genes. Names of *Rpp* genes detected using bulked segregant analysis are in brackets

^c^ Seedling reactions to a mixture of four foreign *P. pachyrhiz*i isolates in greenhouse assays conducted by Miles et al. (2006) before soybean rust had been reported in the continental United States: susceptible (S), resistant (R), or mixed infection types indicative of differential reactions to the isolates
